# Functional Motor Recovery from Motoneuron Axotomy Is Compromised in Mice with Defective Corticospinal Projections

**DOI:** 10.1371/journal.pone.0101918

**Published:** 2014-07-08

**Authors:** Yuetong Ding, Yibo Qu, Jia Feng, Meizhi Wang, Qi Han, Kwok-Fai So, Wutian Wu, Libing Zhou

**Affiliations:** 1 Guangdong-Hongkong-Macau Institute of CNS Regeneration, Jinan University, Guangzhou, P.R. China; 2 Medical Key Laboratory of Brain Function and Diseases, Jinan University, Guangzhou, P.R. China; 3 Department of Anatomy LKS Faculty of Medicine, The University of Hong Kong, Hong Kong, P.R. China; 4 Department of Pathophysiology, School of Medicine, Jinan University, Guangzhou, P.R. China; 5 Co-Innovation Center of Neuroregeneration, Nantong University, Jiangsu, P.R. China; Inserm, France

## Abstract

Brachial plexus injury (BPI) and experimental spinal root avulsion result in loss of motor function in the affected segments. After root avulsion, significant motoneuron function is restored by re-implantation of the avulsed root. How much this functional recovery depends on corticospinal inputs is not known. Here, we studied that question using *Celsr3|Emx1* mice, in which the corticospinal tract (CST) is genetically absent. In adult mice, we tore off right C5–C7 motor and sensory roots and re-implanted the right C6 roots. Behavioral studies showed impaired recovery of elbow flexion in *Celsr3|Emx1* mice compared to controls. Five months after surgery, a reduced number of small axons, and higher G-ratio of inner to outer diameter of myelin sheaths were observed in mutant versus control mice. At early stages post-surgery, mutant mice displayed lower expression of GAP-43 in spinal cord and of myelin basic protein (MBP) in peripheral nerves than control animals. After five months, mutant animals had atrophy of the right biceps brachii, with less newly formed neuromuscular junctions (NMJs) and reduced peak-to-peak amplitudes in electromyogram (EMG), than controls. However, quite unexpectedly, a higher motoneuron survival rate was found in mutant than in control mice. Thus, following root avulsion/re-implantation, the absence of the CST is probably an important reason to hamper axonal regeneration and remyelination, as well as target re-innervation and formation of new NMJ, resulting in lower functional recovery, while fostering motoneuron survival. These results indicate that manipulation of corticospinal transmission may help improve functional recovery following BPI.

## Introduction

Brachial plexus injury (BPI) is a neurological complication of shoulder trauma, most commonly due to vehicle, especially motorbike accidents [1]. Severe forms lead to avulsion of nerve roots, with loss of motoneurons, muscle denervation, atrophy and dysfunction [2]. A model to assess recovery following BPI is the experimental avulsion of nerve roots followed by their re-implantation. Root re-implantation or peripheral nerve grafts help injured motoneurons to survive, regrow axons and re-innervate target muscles, which contributes to functional recovery [3]–[6]. Limb motor function is directly controlled by spinal motoneurons, the activity of which is regulated by cortical input, mainly via the CST [7]–[9]. In rodents, CST axons reach the brainstem-spinal cord junction around birth (P0), and the refinement of their projections is complete ten days later (P10) [10]–[12]. During that P0-P10 interval, several changes occur in the spinal cord. Muscle afferent synapses to spinal motoneurons are gradually eliminated [13], [14] and the strength of the monosynaptic stretch reflex decreases [15]. The inner circuitry in the spinal cord is rearranged; the distribution of spinal interneurons is refined and inactivation of the corticospinal system hampers this reorganization [16]. Expression of genes such as c-Jun and Parvalbumin (PV), fluctuates in parallel with variations in cortical input, and early inhibition or lesion of the motor cortex perturbs their expression pattern in the spinal cord [17]–[19]. In mice with an isolated cortex, the spinal cord, particularly motor components, cannot mature completely [20]. In contrast, in the adult, motor cortex lesions do not induce much reaction in the spinal cord [19], indicating that spinal cord maturation proceeds during a critical period, after which plasticity is compromised.

Whether motoneuron recovery following BPI is regulated by cortical input remains poorly understood. Previous studies focused on the effect of cortical stimulation on the regeneration of corticospinal axons. In rat CST lesion models, electrical stimulation of spared corticospinal axons enhances their sprouting and strengthens connections with spinal motor circuits [21]. Chronic electrical stimulation of the intact motor cortex promotes the growth of corticospinal axon to the denervated side of the spinal cord and brain stem, which contributes to locomotor restoration [22], [23]. In humans with chronic spinal cord injury, strengthening corticospinal synaptic transmission using transcranial magnetic stimulation promotes motor recovery [24].

To assess the impact of corticospinal input on motoneuron recovery, we compared motoneuron reaction, axonal regeneration and motor function after brachial plexus avulsion and root re-implantation in control and in *Celsr3|Emx1*mutant mice, in which the CST is congenitally deficient [25]. Our results indicate that modulation of cortical input maybe regulate motoneuron axonal regeneration, hinting at possible future applications.

## Materials and Methods

### Ethics statement

Animal procedures were performed in strict accordance with the recommendations in the Guide for the Care and Use of Laboratory Animals of the National Institutes of Health. The protocol was approved by Laboratory Animal Ethics Committee at Jinan University (Permit Number: 20111008001). All surgery was performed under avertin anesthesia, and all efforts were made to minimize the suffering and number of animals used.

### Animals

We crossed [Celsr3+/-; Emx1-Cre] males with Celsr3f/f females to obtain [Celsr3f/-; Emx1-Cre] mutant mice (*Celsr3|Emx1* for short), in which Celsr3 is conditionally inactivated in Emx1 positive cells, and used [Celsr3f/+; Emx1-Cre] as controls. The following primers were used for genotyping. Celsr3 18S: 5′-AGC CAA GAT GTC CGA GTC AC-3′, Celsr3 19AS: 5′-GCC CAC AAG TGT CCT GTC TC-3′, Celsr3 28AS: 5′-AGC ATG GAG GTA GTG GAA GG-3′; Emx1_IRESF: 5′-GCG AGC CTT TGA GAA GAA TC-3′, Emx1_RESR: 5′-CCT TAT TCC AAG CGG CTT CG-3′ [25].

### Brachial plexus avulsion and re-implantation models

**Figure 1:**
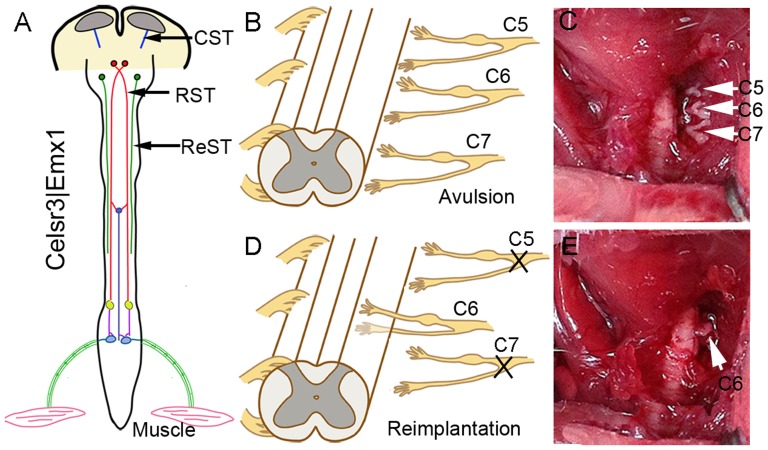
Animal model. A: In *Celsr3|Emx1* mice, corticospinal axons (CST) do not reach the internal capsule and the spinal cord, but the rubrospinal (RST) and the reticulospinal tracts (ReST) are preserved. B–E: Surgical root avulsion/re-implantation procedure. At the cervical enlargement, right C5–C7 ventral and dorsal roots are tore off (B, arrows in C), the proximal endings of C5 and C7 are severed, and C6 roots are re-implanted to the corresponding segment (D, arrow in E).

Adult mutant and control mice (2–3 month old, weight 25–30 g) were anaesthetized with avertin (intraperitoneal injection, 13 µl/g). Following hemilaminectomy, the dura mater was opened and C5–C7 segments were identified based on their location relative to the long spine of T2. Right C5–C7 dorsal and ventral roots were dissected out, the 2–3 mm proximal segments of C5 and C7 were cut off to prevent reconnection to the spinal cord, and the right C6 ventral root was inserted back to its original location (Fig. 1). Following recovery from surgery, animals were housed in individual cages. They resumed drinking and eating within 1 day and recovered uneventfully. They were monitored and studied for up to 5 months after surgery.

### Behavioral studies

Behavioral tests were carried out by an experimenter blind to mouse genotypes.

#### Grooming test

**Figure 2:**
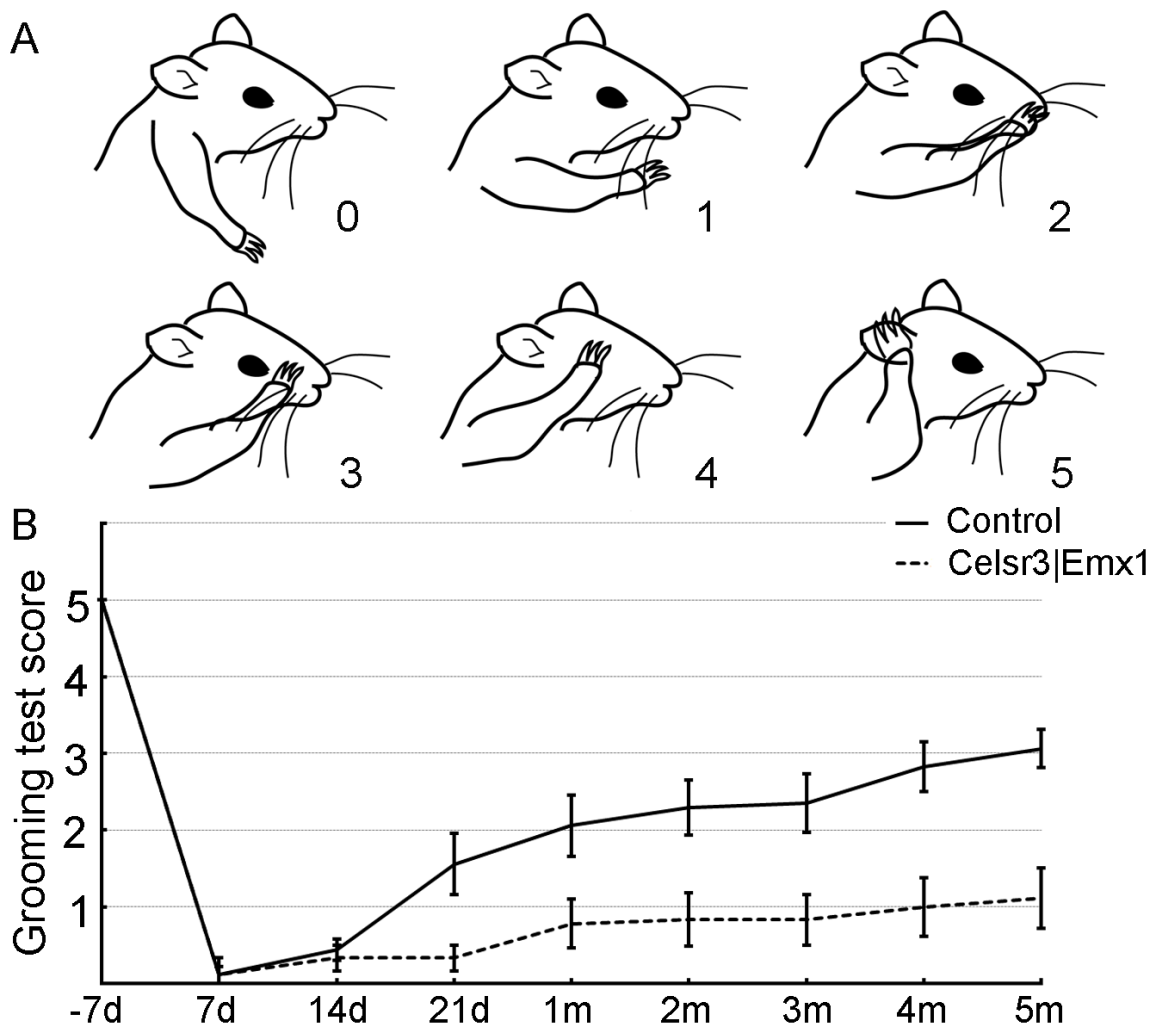
*Celsr3|Emx1* mice show poor recovery of elbow flexion after surgery. A: Positions of forelimbs and corresponding scores in grooming tests (adapted from Bertelli & Mira, 1993). B: Mean scores of grooming tests pre- and post-surgery in both groups. Before surgery, the mean score around 5 was comparable in both genotypes. Control mice initiated a progressive recovery around 21 days, reaching a mean score of 3.11±0.25 after five months. In the mutant group, a few mice showed functional recovery from one month post surgery, but the mean score from day 21 to 5 months was lower than in control mice (*t*-test, *P*<0.01, n = 12).

Elbow flexion was evaluated using the grooming test [26]. Briefly, water was sprayed on the animal's head, and forelimb movements to remove the water were recorded with a video camera for 2 min. Based on the position of the forepaw upon elbow flexion, the score ranged from 0 to 5 as follows: 0, no response; 1, elbow flexion without touching the nose; 2, touching the nose; 3, reaching below the eye; 4, reaching the eye; 5, reaching the ear or back of the ear (Fig. 2A).

#### Catwalk analysis

Walking was assessed 5 months post surgery, using the Catwalk system and the EthoVision XT 9.0 software (Noldus, The Netherlands). The conditions for collecting data were to complete one-way walk in 1–10 sec, with a walking speed variation less than 60%. Footprint patterns, maximal contact area (foot to floor area) and intensity of contact were measured. Footprint Intensities were recorded by plotting print intensities of 4 paws in one individual frame.

### Electromyography (EMG)

The function of biceps brachii was estimated by EMG [20]. Briefly, the musculocutaneous nerve and biceps brachii were partially exposed under a surgical microscope under avertin anesthesia. A stimulating bipolar electrode was placed on the musculocutaneous nerve and a recording electrode was inserted in the center of the biceps, with a grounding electrode in the subcutaneous tissue. Similar stimulations (0.3 mA, 0.5 ms, 1 Hz) were applied for 20 ms in all animals. EMG signals were collected with a multi-channel signal acquisition and processing system (RM6240BD, Chengdu, China). Individual responses were measured three times, at 2-min intervals, on left and right sides. The peak-to-peak amplitude of evoked potentials was measured and expressed as the ratio between the operated (R) and the unoperated (L) sides. After recording, the biceps brachii were fixed in 4% paraformaldehyde to estimate the R/L weight ratio.

### Histology and immunohistochemistry

Thirty µm-thick serial frozen sections were stained with 0. 1% cresyl violet to visualize tissue morphology. For immunohistochemistry, sections were washed in 0.1 M phosphate buffer saline (PBS), blocked in 10% goat serum and 3% albumin bovine serum for 2 hr, and incubated with primary antibodies overnight at 4°C. Signal was detected with Alexa fluor 546 or 488 fluorescent secondary antibodies (1∶1000, Invitrogen). Primary antibodies were: goat anti-choline acetyl transferase (ChAT, 1∶500, AB144p, Millipore), rat anti-CD11b (1∶1000, MCA711G, AbD serotec), rabbit anti-GAP-43 (1∶2000, AB5312, Chemicon), mouse anti-NeuN (1∶1000, MAB377, Chemicon), rabbit anti-MBP (1∶200, M3821, Sigma), rabbit anti-c-Jun (1∶500, Cat. No. 9165, CST) and mouse anti-Parvalbumin (PV, 1∶2000, Cat. No. MAB1572, Millpore).

### NMJs morphology

Five months after surgery, forty µm-thick frozen longitudinal sections of the biceps brachii were prepared using a sliding microtome (Leica SM 2010R). Rabbit anti-neurofilament 200 (NF200, 1∶1000, N4142, Sigma) and alpha-bungarotoxin conjugated to Alexa fluor 546 (*a*-BT, 1∶1000, T1175, Molecular Probes) were used to label axons and acetylcholine receptors (AchRs). For each section, five isolated AchR clusters were photographed under100× oil objective and 100 NMJs were studied in 4 animals in each group. NMJs were ranked in three categories: mature, denervated and immature (remodeled and neoformed) as described [27], and the proportion of each type was calculated.

### Assessment of regeneration and remyelination

Following 4% paraformaldehyde perfusion, 4 mm-long proximal, middle and distal portions of musculocutaneous nerves were collected on days 7, 14 and 21, and post-fixed at 4°C overnight. Nerve segments were divided in two for preparation of longitudinal and transverse sections. Seven µm-thick cryostat sections were used for MBP immunofluorescence. For each transverse section, the fluorescence intensity within myelin sheath was estimated using Image J, and the mean of proximal, middle and distal portions (each portion containing 5 sections) was estimated to represent one sample. Four animals were used in each group.

Five months after surgery, mice were anesthetized with avertin and perfused intracardially with a solution of 2% glutaraldehyde and 2% paraformaldehyde in 0.15 M phosphate buffer (PB, pH7.4). One mm-long middle segments of musculocutaneous nerves were immersed in the same fixative overnight at 4°C, washed in 0.15 M PB, and postfixed in 0.5% osmium tetroxide for 1–2 hr. Following dehydration in alcohol and embedding in resin (EMbed 812, Electron Microscope Sciences), small samples (about 1 mm) were glued in resin blocks. 700 nm-thick transverse sections were stained with1% Toluidine Blue for imaging under a 100× oil objective. For each section, the number of differently sized axons and G-ratio was measured. The G-ratio, the ratio of the inner to the outer diameter of the myelin sheath, is widely used to assess axonal myelination. The normal value is about 0.6 in normal peripheral nerves [28]. During regeneration, axons undergo an initial period of hyper-remyelination with low G-ratio, which then reverts gradually to the normal value [28]. Three animals were used in each group.

### Estimation of cell numbers and measuring fluorescence density in spinal cords

To study changes of spinal motoneurons in the cervical enlargement, serial sections from C5–C7 segments, prepared 5 months post surgery, were allocated to 4 alternative series of 10 sections. One series was stained with 0.1% cresyl violet (Nissl stain), one with anit-NeuN and another one with anti-ChAT. Nissl-stained motoneurons were identified as described [29], large NeuN-positive and ChAT-positive neurons were counted in the ventral horn. The number of cells was estimated separately on the left (L) and right (R) side of each section. The mean from one series of section was taken as one sample and the R/L ratio was calculated. Six animals were used in each group.

To calculate c-Jun and Parvalbumin (PV) positive cells in the ventral horn, we prepared serial sections from C5–C7 segments 3 days post surgery and immunostained alternative series of about 10 sections for each antibody. The number of c-Jun or PV positive cells in the ventral horn was counted separately on the left (L) and right (R) sides of each section. The mean from one series of section was taken as one sample and the R/L ratio was calculated. In each group, 6 animals were used for anti-c-Jun immunostaining and 3 animals were used for anti-PV immunostaining.

Because of GAP-43 expression only in the injured ventral horn and CD11b expression on the whole injured side after injury, we captured images of anti-GAP-43 stained sections under 20× objective containing the ventral horn and of anti-CD11b stained sections under 10× objective containing the right hemi-segment. Twelve images were taken from each animal for each staining and Integrity Density of fluorescence was measured by Image J. with a auto-set and constant threshold to keep the positive signal only. Mean of Integrity Density from 12 images represented the expression level of one animal and results were compared with one-way ANOVA. Four animals were used at each time-point of each group.

### Western blot

Three days post surgery, injured and intact sides of C5–C7 hemi-segments were separately collected and homogenized in lysis buffer (20 mM Tris-HCl pH7.5, 150 mM NaCl, 1% Triton X-100, 25 mM NaPPi, 80 mM *β*-glycerophosphate, 2 mM EDTA, 0.2 mM Na_3_VO_4_). Lysates were frozen/thawed four times, and cleared by centrifugation at14,000 rpm for 15 minutes at 4°C. Supernatants were pooled and protein concentrations were measured using the Bradford method. Samples containing 30 µg total protein were analyzed on 10% SDS-PAGE gels and transferred to nitrocellulose membrane (BioScience) by electroblotting (Bio-Rad). Membranes were blocked with 3% BSA and 1% Tween20, in PBS, for 30 min, and incubated with rabbit anti-BDNF (1∶2000, Cat.No. ab6201, Abcam), goat anti-TrkB (1∶2000, Cat.No. AF1494, R&D), rabbit anti-NT-3 (1∶2000, Cat.No. ab65804, Abcam), rabbit anti-GDNF (1∶1000, Cat.No. ab18956,Abcam), rabbit anti-p75(1∶2000,Cat.No. G3231,Promega) and rabbit anti-*β*-tubulin (1∶1000, Cat.No. 2128S, CST), at 4°C overnight. Signal was detected using HRP-conjugated rabbit or goat antibodies followed by chemiluminescence using a Super Signal West Pico kit (Pierce) and Hyper film ECL (Amersham Biosciences). All experiments were carried out at least in triplicate. Autoradiography films were scanned and signals were quantified using Image J. In each experiment, signal of each protein was normalized to the corresponding control protein, *β*-tubulin, and the data were presented as the ratio of each protein to the control protein and analyzed using Student's *t*-test. Six animals were used in each group.

### Statistical analysis

Data are presented as mean ±SEM. Cell numbers, fluorescence density of different markers and the corresponding R/L ratios in control animals were compared with those in *Celsr3|Emx1* animals. Electrophysiological data and muscle weights were compared with those from the control contralateral sides, and/or between control and mutant groups. One-way ANOVA or *t*-test, with a Student–Newman–Keuls test or a Bonferroni correction for multiple comparisons, was used. Results were considered significant (*) at *P*-values <0.05 and highly significant (**) at *P*-values <0.01.

## Results

### Following root avulsion/re-implantation, recovery of forelimb movements is lower in *Celsr3|Emx1* than in control mice

In *Celsr3|Emx1* mutant mice, corticospinal axons do not enter the internal capsule and never reach the spinal cord, a phenotype confirmed using labeling with the Thy1-YFP transgene, PKC_γ_ immunostaining and FluoroGold retrograde tracing [25]. In contrast, the rubrospinal, vestibulospinal and reticulospinal tracts are preserved (Fig. 1A), and mutant mice maintain normal walking abilities, presumably due to preservation of the RST (unpublished observations).

**Figure 3:**
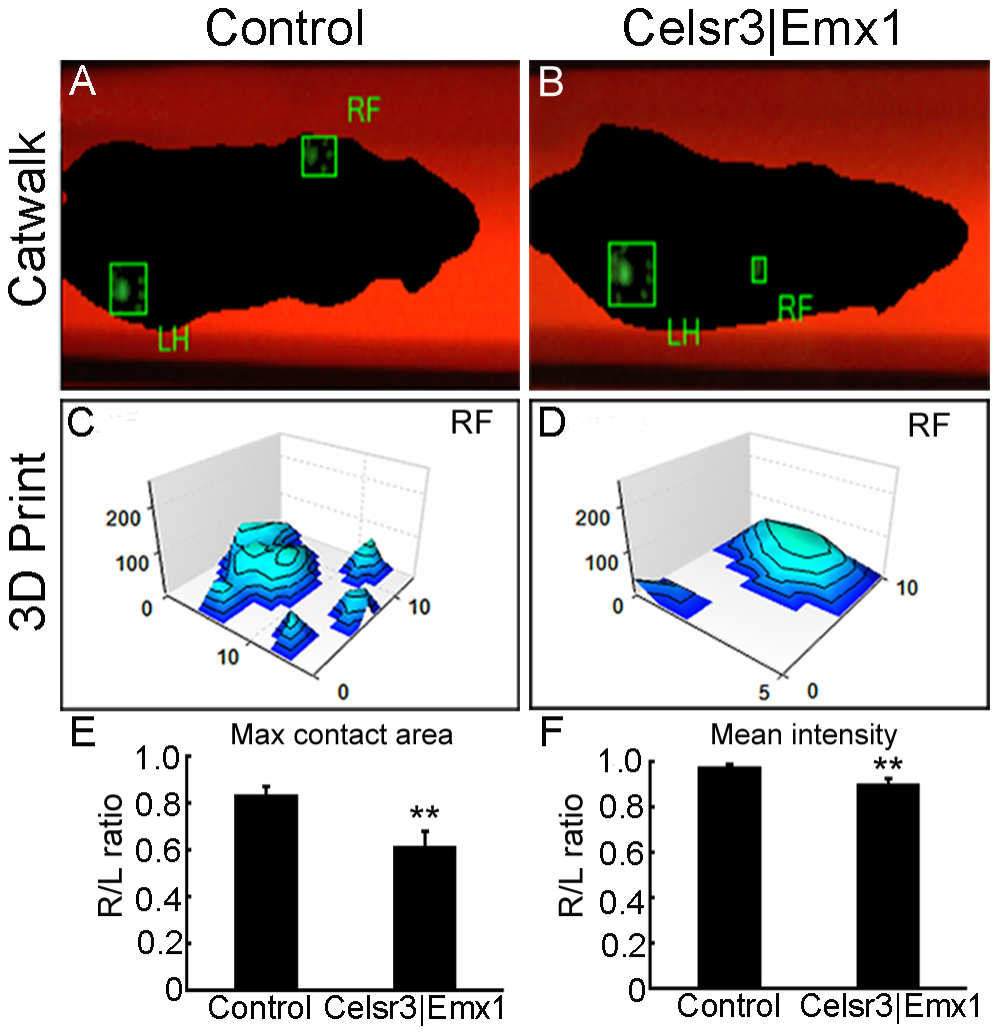
*Celsr3|Emx1* mice display walking deficits after surgery. The walking ability was evaluated using the Catwalk tests five months after surgery. In control mice (A), the footprints (right forelimb, RF and left hind limb, LH) were clearly identified. In mutant mice (B) the RF print of the mutant mouse was blurry, although the intact footprint was distinct. Using tri-dimension (3D) Footprint Intensities charts, control mice showed prints of the palm and finger (C), whereas only palm prints were seen in mutant mice (D). The ratio of maximal contact area of the operated versus intact forepaw (R/L ratio) was significantly decreased in *Celsr3|Emx1* mice (E). Similar results were observed for the R/L mean print intensity ratios (F). **: *P*<0.01, One-way ANOVA, n = 10.

To compare motor recovery in *Celsr3|Emx1* mutant and control mice, we used the root avulsion and re-implantation model (Fig. 1B–E). Since the biceps brachii is mainly innervated by motoneurons from the C6 spinal segment and drives elbow flexion, avulsion of roots C5–7 results in its denervation and elbow flexion disability. Re-implantation of the C6 ventral root provides a bridge that enables motor axons to re-innervate the biceps and restore function [30]. Before surgery, control and mutant mice displayed comparable skills in the grooming test, with a mean score of 5, showing that mice without a CST have normal elbow flexion. After surgery, grooming tests were performed at days 7, 14, and 21, and months 1, 2, 3, 4 and 5. In control mice, recovery began around day 21, and the mean score increased gradually to reach a plateau between months 3 and 5. In contrast, few mutant mice showed any functional recovery, the mean score remaining at 1 until 5 months post surgery (Fig. 2B). Gait was studied using Catwalk tests at 5 months post-surgery. In controls, prints of the right forelimb, including the palm and five paws, were easily identified (Fig. 3A). By contrast, prints of mutant right forelimbs were blurred, although hindlimb prints were comparable to controls (Fig. 3B). In tridimensional (3D) cumulative prints, the paws and palms were easily identified in the control but not in the mutant (Fig. 3C, D). We measured the maximal contact area and the mean intensity of the right (R, surgery side) and the left (L, unoperated side) forelimbs and used the R/L ratio as an index of walking recovery. The R/L ratio of maximal contact areas was 0.84 in the control group, versus 0.61 in the mutant, a significant difference (Fig. 3E; *P*<0.01, n = 10). Similarly, the R/L ratio of the mean intensity was significantly lower (0.89) in mutants than in control animals (0.98) (Fig. 3F; *P*<0.01, n = 10).

### More spinal motoneurons survive after root avulsion in *Celsr3|Emx1* than in control mice

**Figure 4:**
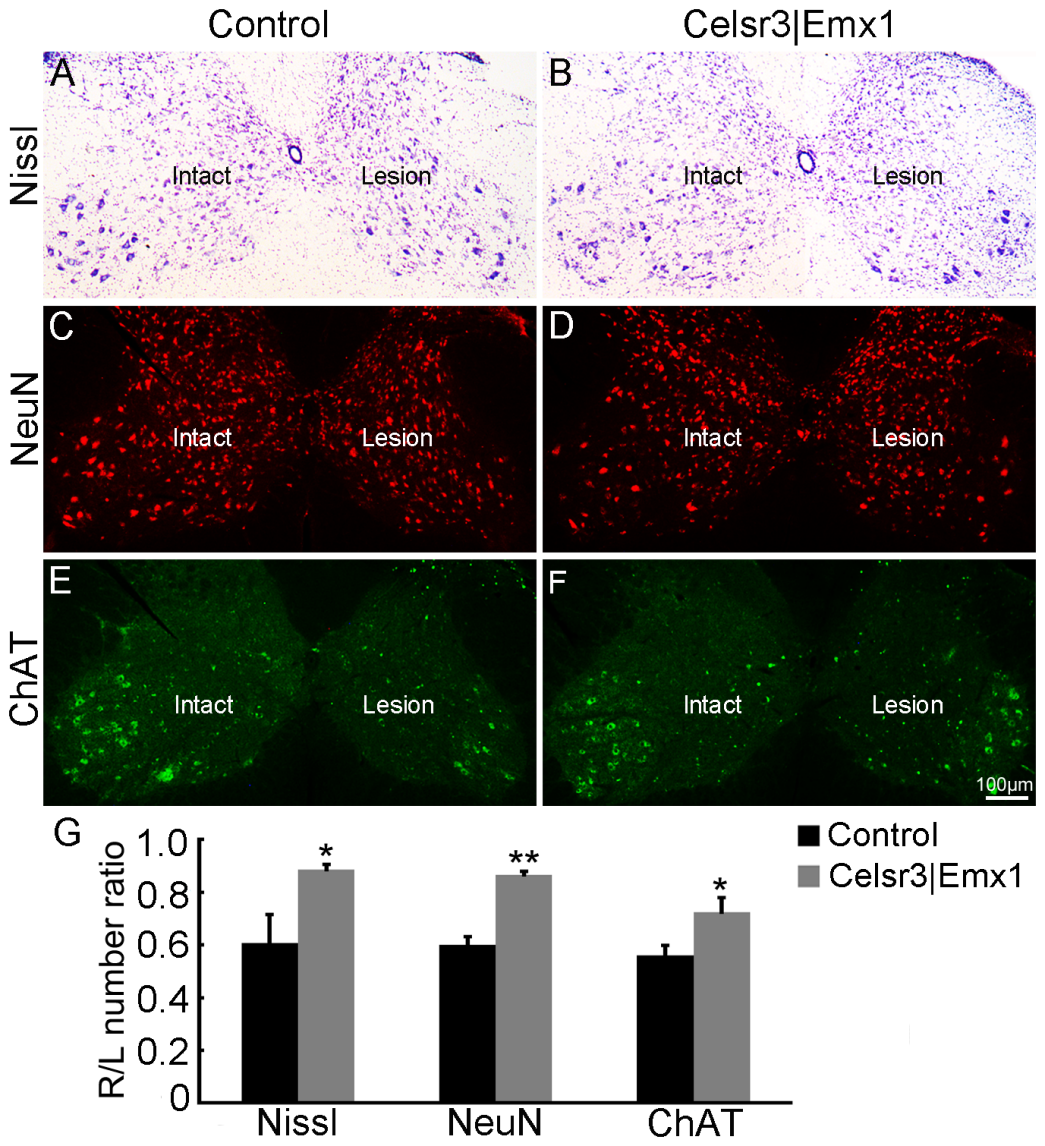
Spinal motoneurons are more resistant to the avulsion injury in *Celsr3|Emx1* than control mice. C5–C7 segments of spinal cords were collected 5 months post surgery for Nissl staining, anti-NeuN and anti-ChAT immunofluorescence. A–F: Transversal sections at C5–C7 in control (A, C, E) and mutant spinal cord (B, D, F), stained with Nissl (A, B), anti-NeuN (C, D) and anti-ChAT (E, F). Differences in neuron numbers between Intact and Lesion sides are evident and quantified in G, using the Intact/Lesion (R/L) ratios as indexes. *, *P*<0.05; **: *P*<0.01, *t*-test, n = 6 in each group.

**Figure 5:**
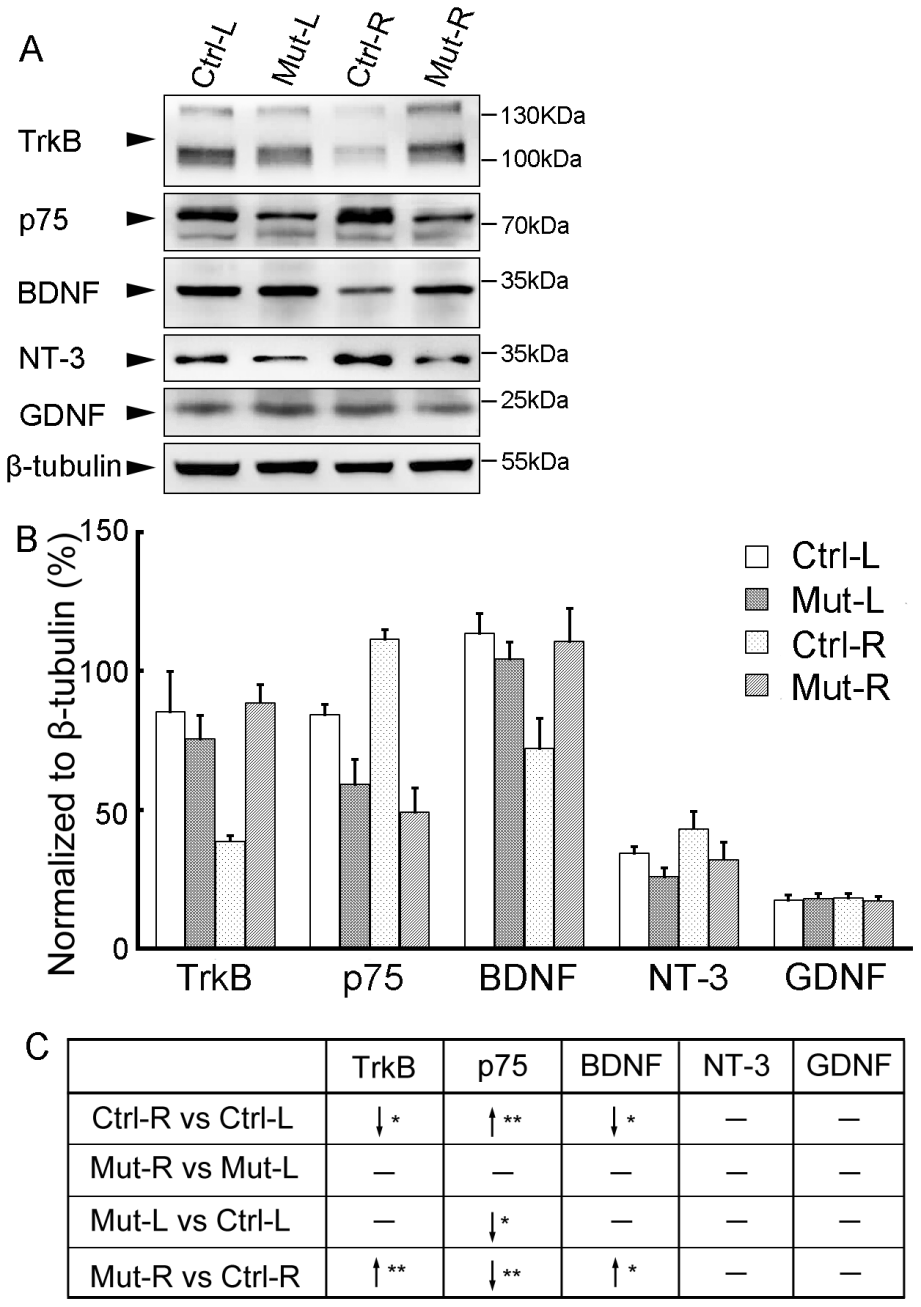
Expression profile of neurotrophic factors and receptors 7 days post surgery. Seven days post surgery, injured (R) and intact (L) sides of C5–C7 segments were collected separately for Western blot with anti- TrkB, p75, BDNF, NT-3 and GDNF antibodies in control (Ctrl) and *Celsr3|Emx1* (Mut) mice, and *β*-tubulin was used as a control protein (A). The expression level of each protein was normalized to*β*-tubulin (B). Comparisons were summarized in C. In control mices, the expression of TrkB and BDNF was significantly decreased, but that of p75 was significantly increased, on injured sides (R) compared to intact sides (L). There was not significant difference in expression of neurotrophic factors and receptors between injured and intact sides in mutant mice. On both sides, the expression level of p75 was significantly lower in the mutant than in the control. On injured sides, the expression of TrkB and BDNF was significantly higher in the mutant than in the control although their expression was comparable on intact sides between two groups. “↓”, lower or decreased expression; “↑”, higher or increased expression; “_”, no significant difference; *, *P*<0.05; **, *P*<0.01; Ctrl-L, left side of control mice; Ctrl-R, right side of control mice; Mut-L, left side of mutant mice; Mut-R, right side of mutant mice. Six animals were used in each group and *t*-test was used for comparisons.

Five months after surgery, right C5–C7 spinal segments showed signs of shrinking in both mutant and control mice. To compare motoneuron survival, we estimated motoneuron numbers in the ventral horn on lesioned (right, R) and unlesioned (left, L) sides, using Nissl staining. In both groups, the number of motoneurons was decreased on the lesioned compared to the unoperated side (Fig. 4A, B). However, the R/L ratio of motoneuron number was 61% in the control and 89% in the mutant, a significant difference (Fig. 4G; *P*<0.05, n = 6 in each group). A similar result was obtained with anti-NeuN staining, 60% in controls versus 87% in mutants (Fig. 4C, D, G; *P*<0.01, n = 6 in each group). We confirmed that result using anti-ChAT immunofluorescence, which labels spinal motoneurons in the ventral horn and a few interneurons around the central canal [20]. On the lesioned side, ChAT-positive motoneurons were much reduced in number in both groups (Fig. 4E, F), with R/L ratios of 56% in the control and 72% in the mutant, a significant difference (Fig. 4G; *P*<0.05, n = 6 in each group). Motoneurons were less numerous in mutant than in control unlesioned spinal cord, thereby emphasizing the difference observed in the R/L ratios, and indicating that mutant motoneurons were more resistant to the avulsion lesion than control ones. As neurotrophic factors play important roles in influencing neuronal survival [31], we compared the expression of TrkB, p75, BDNF, NT-3 and GDNF with Western blot 7 days post surgery (Fig. 5). In control mice, the expression of TrkB and BDNF was significantly decreased on injured sides compared to intact sides. However, there was not much diiference of neurotrophic factors and receptors between two sides in mutant mice, which resulted in comparably higher expression of TrkB and BDNF on injured sides in the mutant than in the control although their expression was comparable on unoperated sides between two groups. The surgery induced an increase of p75-expression on the injured side in control mice and there was a lower expression on both sides in mutant mice compared to control mice. Thus, the changes in expression levels of neurotrophic factor and receptors post surgery are probably associated with different motoneuron survival rates between the two genotypes.

**Figure 6:**
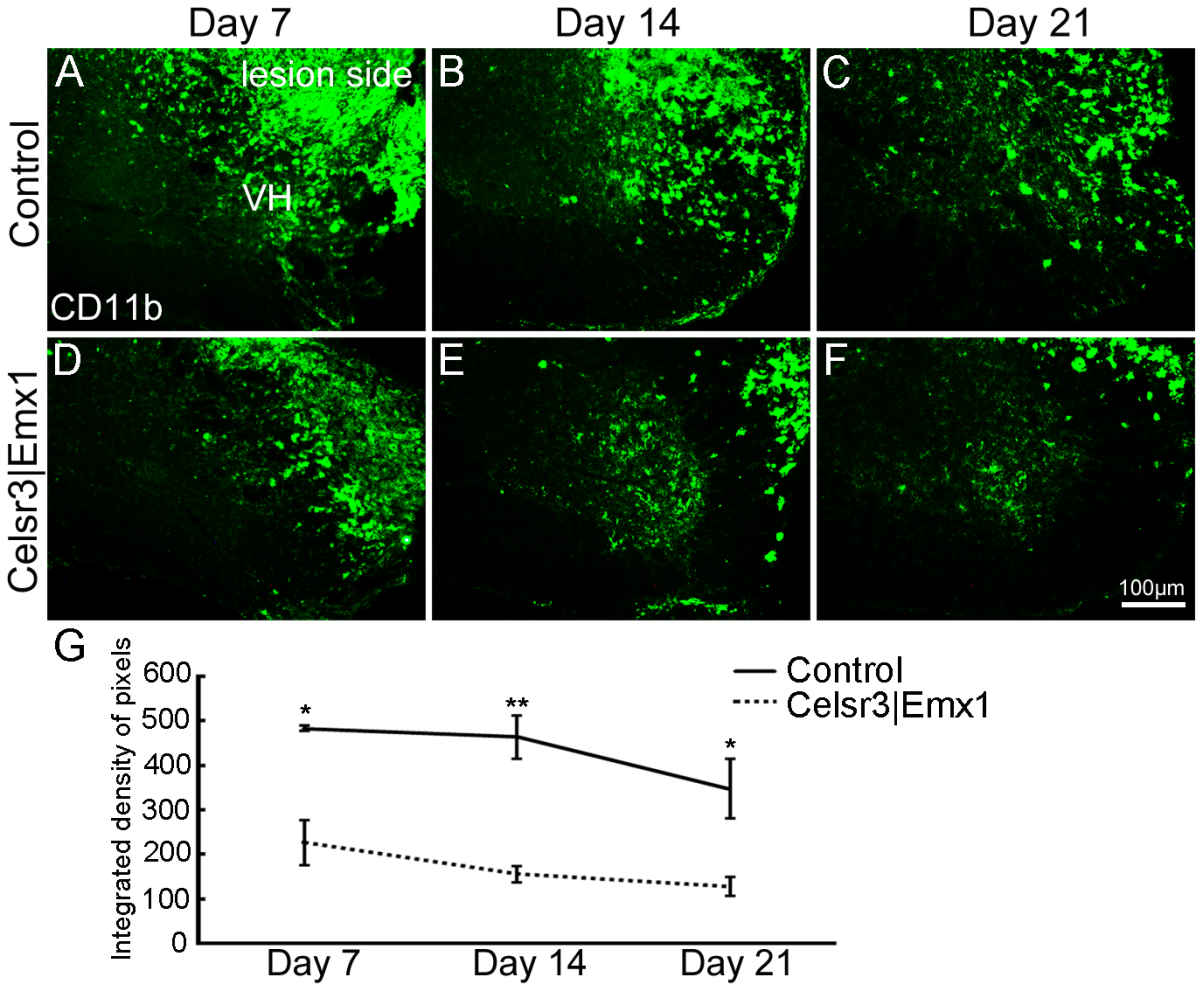
Macrophages are activated followed root avulsion/re-implantation. In transverse sections at C5–C7, anti-CD11b immunoreactivity is widely distributed in the white and gray matter on the lesion side with a decreasing trend from day 7 to day 21, in both the control (A–C) and the mutant (D–F). At each time-point, CD11b expression is higher in the control than the mutant (G). VH, ventral horn. *, *P*<0.05; **, *P*<0.01; comparison at different time-points with *t*-test, n = 4 mice in each group.

Root avulsion activates macrophages, presumably to remove injured cells [2]. To detect activated macrophages, we used anti-CD11b immunofluorescence staining on days 7, 14 and 21 post surgery. CD11b positive cells were found on the lesioned but not the unoperated side. On day 7, intense CD11b-ir was widely dispersed in the ventral and dorsal horns, and in the white matter in both groups, albeit less intense in mutant than in control samples (Fig. 6A, D). It decreased during subsequent days to reach a minimal level on day 21 (Fig. 6B, C, E, F). At all time points, CD11b-ir was lower in mutants than in controls (Fig. 6G). Less macrophage reactivity in the mutant may help explain the higher rate of motoneuron survival and the slower regeneration of their axons, leading to reduced functional recovery.

**Figure 7:**
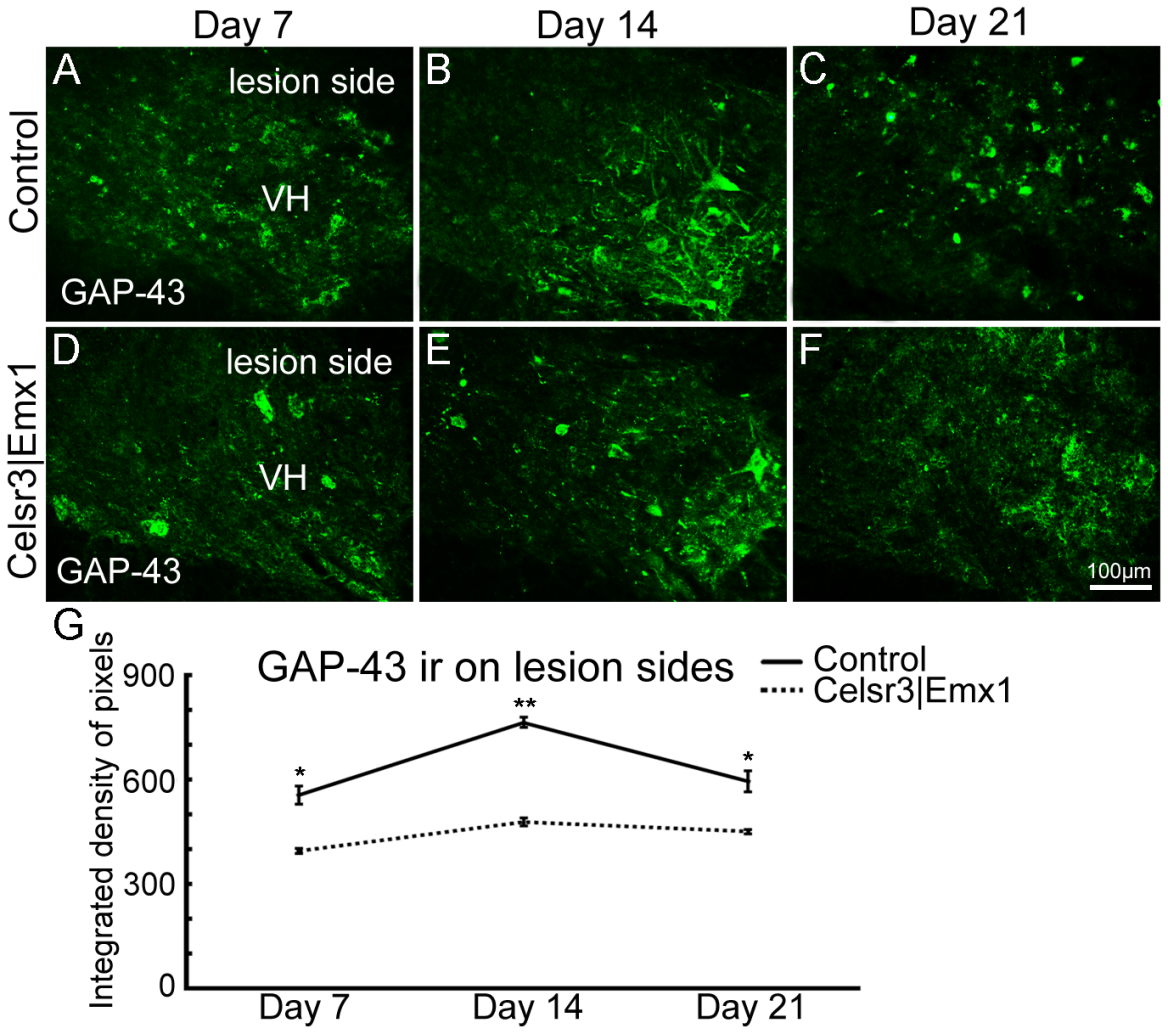
Regeneration estimated by GAP-43 expression in the post surgery spinal cord. In transverse sections at C6, GAP-43 immunoreactivity (ir) is concentrated in the ventral horn (VH) on the lesion side on days 7, 14 and 21 post surgery, reaching maximal level on day 14, in both the control (A–C) and the mutant (D–F). At each time-point, GAP-43 expression is higher in the control than the mutant (G). *, *P*<0.05; **, *P*<0.01; comparison at different time-points with *t*-test, n = 4 mice in each group.

**Figure 8:**
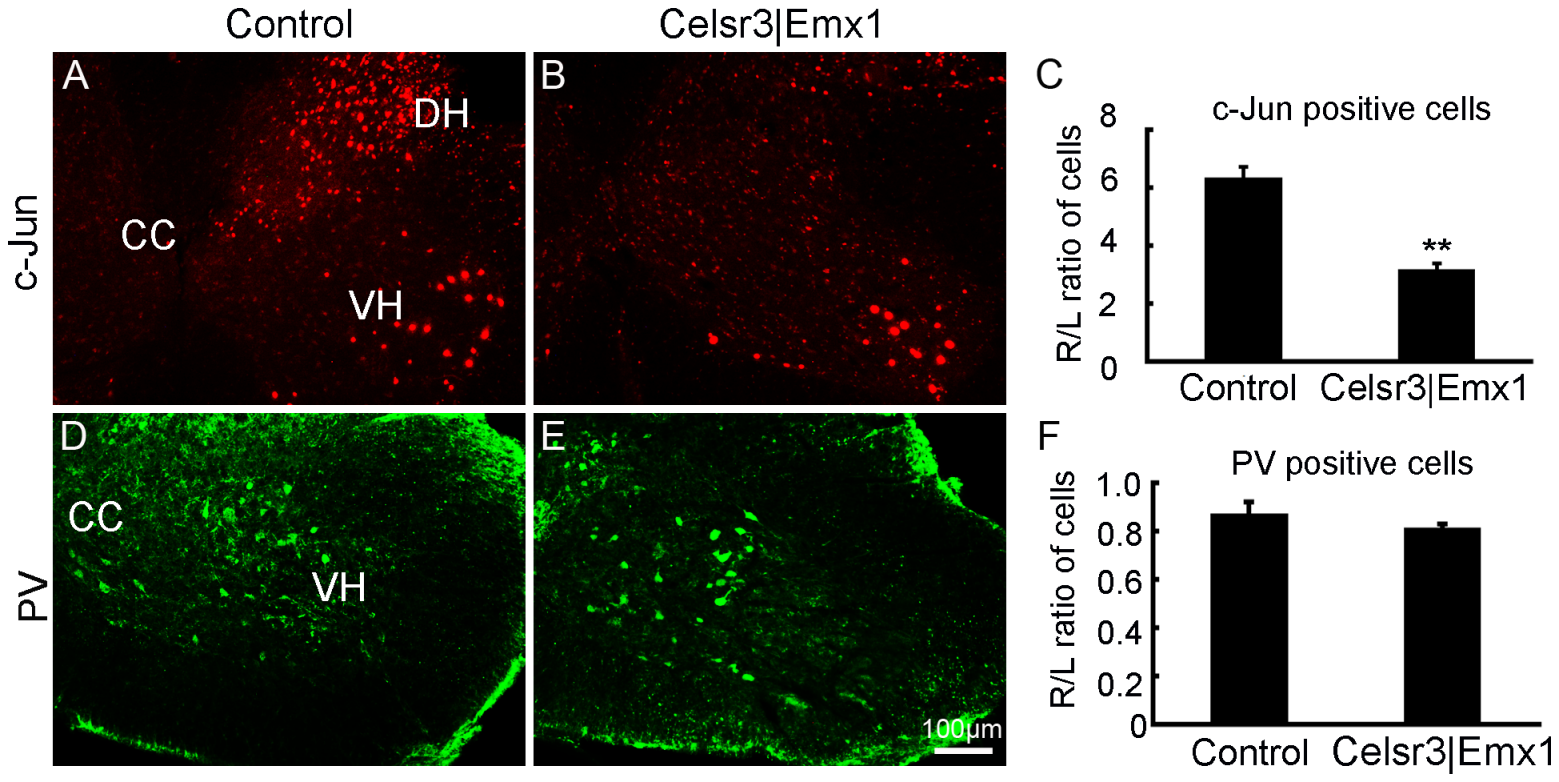
Expression of c-Jun, not Parvalbumin, is increased at the early stage of post-surgery. C5–C7 spinal segments were prepared for anti-c-Jun and Parvalbumin (PV) immunofluorescence 3 days post surgery. The expression of c-Jun was rapidly increased on injured sides in both groups, mostly in the ventral horn (VH) and the dorsal horn (DH) (A, B). The number of c-Jun positive cells was counted in the VH on both sides, and its ratio of the right side to the left side (R/L) was calculated, showing significant lower in the mutant than in the control (C). PV- positive neurons were easily identified in the middle region of the gray matter (D, E), but the R/L ratio of the cell number showed no statistic difference between two genotypes (F). CC, the central canal; **, *P*<0.01; n = 6 animals in each group for c-Jun immunostaining and n = 3 animals in each group for PV immunostaining; comparison by *t*-test.

To estimate the regeneration ability of surviving neurons, we studied GAP-43 expression at days 7, 14 and 21. At the level of C6, GAP-43 immunoreactivity (ir) in ventral horns was high on the side of the lesion, but absent on the intact side. From days 7 to 14, the intensity of GAP-43-ir increased gradually, and then decreased in both groups (Fig. 7A–F). However, GAP-43 expression was significantly lower in mutants than in controls at all three time-points (Fig. 7G), indicating that surviving motoneurons in the control may have stronger regeneration potential. As described before, the expression of c-Jun and PV fluctuates accompanying with variations in cortical input. As an immediate-early gene, c-Jun acts as an important regulator of axonal regeneration in the injured central nervous system [32] and it is expressed in spinal motoneurons after root avulsion [33]. We then studied their expression 3 days post surgery (Fig. 8). In injured ventral horns, c-Jun positive cell number was about 5-fold increase in control mice and 2-fold increase in mutant mice compared to intact ventral horns, and the R/L ratio of c-Jun positive cells in the mutant was significantly lower than in the control (Fig. 8A–C, *P*<0.01, n = 6), which could contribute to more efficient axonal regeneration in the control than in the mutant. However, the expression change of PV was comparable between two groups (Fig. 8D–F, *P*>0.05, n = 3).

### Motor axon regeneration and remyelination are delayed in *Celsr3|Emx1* mice

**Figure 9:**
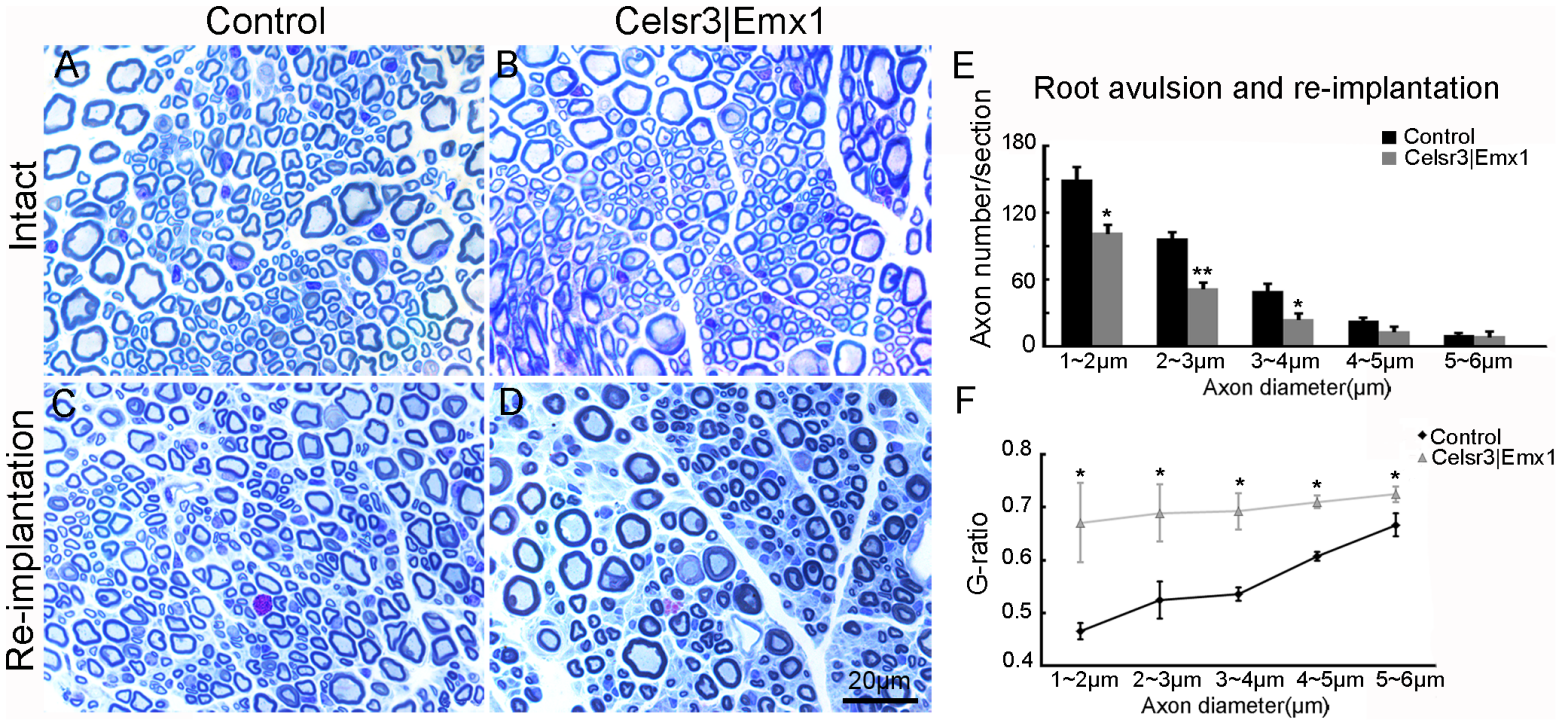
Post surgery axon number and G-ratio are different in the two groups. Unlesioned, normal nerves (A, B) are similar in both genotypes. Five months after root avulsion/re-implantation (C, D), smaller axons are more abundant in control than in mutant regenerating nerves (quantification in E), and the G-ratio (the ratio of the inner to the outer diameter of the myelin sheath) is higher in mutant than control samples (quantified in F). A–D: Plastic semi-thin sections stained with Toluidine Blue. *: *P*<0.05; **: *P*<0.01; comparison at different sized axons with *t*-test, n = 3 mice in each group.

To evaluate axonal regeneration and remyelination, we examined musculocutaneous nerves at 5 months. In intact nerves, the number of axons and their diameters were comparable in both genotypes (Fig. 9A, B). In regenerating nerves, the number of small axons (diameter 1–4 µm) was significantly decreased, whilst the number of larger axons (the diameter>4 µm) was similar in *Celsr3|Emx1* versus control samples (Fig. 9C, D, E), indicating that mutant nerves contained less regenerating axons (n = 3 animals in each group). In controls, the G-ratio of small axons (diameter<4 µm) was less than 0.55, indicating axon hyper-myelination, whereas that of larger axons (diameter>4 µm) was about 0.6, suggesting maturation. In contrast, an elevated G-ratio (more than 0.65) was found for all axon categories in the mutant (Fig. 9F), suggesting that impaired remyelination affected axons of all sizes.

**Figure 10:**
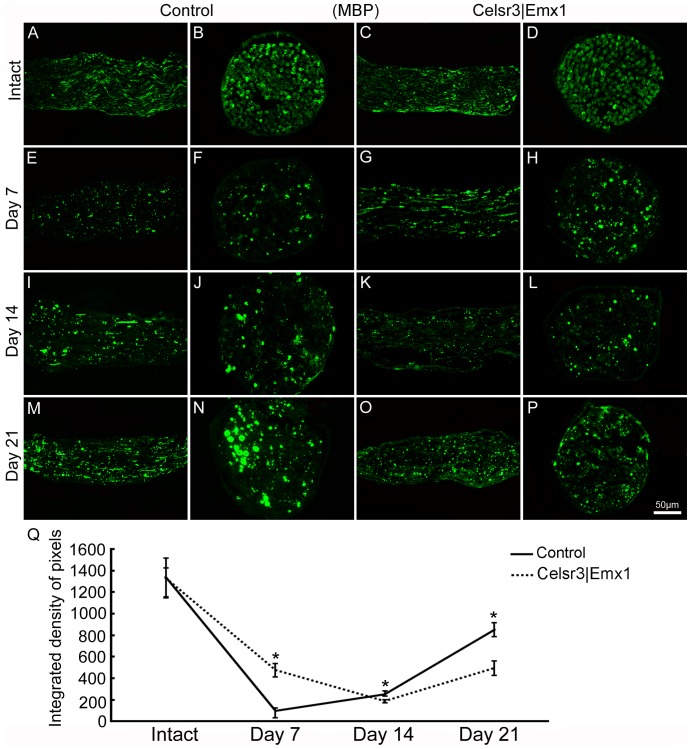
Remyelination is less prominent in *Celsr3|Emx1* than control mice after root avulsion/re-implantation. Longitudinal and transverse sections of musculocutaneuous nerves, prior to, and at different time-points post surgery, stained with anti-MBP. MBP is highly expressed in intact musculocutaneous nerves, with no difference between genotypes (A–D). After surgery, MBP expression decreases and reaches minimal expression after 7 days in the control and 14 days in the mutant, and then increases gradually (E–P). Q: In mutant nerves, MBP density is significantly higher than in control nerves at 7 days but lower after 14 and 21 days. *: *P*<0.05, comparison at different time-points with *t*-test, n = 4 in each group.

To appreciate the dynamics of remyelination, we examined MBP expression in musculocutaneous nerves on days 7, 14 and 21. In both longitudinal and transverse sections, MBP was expressed in intact musculocutaneous nerves, with no difference between genotypes (Fig. 10A–D). After surgery, MBP expression decreased somewhat earlier in the control than in the mutant, reaching a minimal level after 7 days in the control versus 14 days in the mutant. It then increased gradually, still earlier in the control than in the mutant (Fig. 10E–P). Compared to the control, MBP expression was significantly lower in the mutant on day 7, but significantly higher on days 14 and 21 post surgery (Fig. 10Q).

### Absence of the CST hinders new NMJ formation and functional recovery after root avulsion and re-implantation

**Figure 11:**
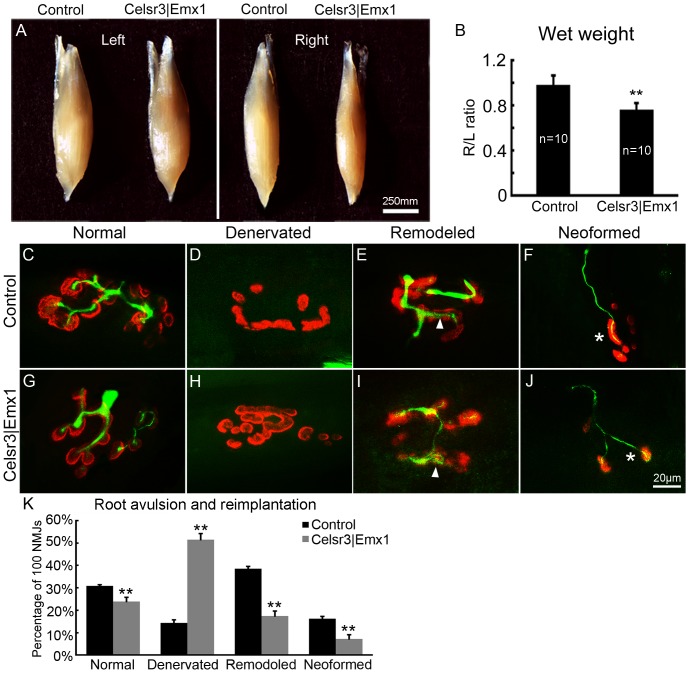
After surgery, newly innervated NMJs are less abundant, and muscles more atrophic in *Celsr3|Emx1* than control mice. A, B: Five months post surgery, left (L, intact side) and right (R, surgery side) biceps brachii were weighted. The R/L wet weight ratio was 97% in control mice versus 80% in mutant mice (**: *P*<0.01, *t*-test, n = 10 in each group). C–J: Different classes of NMJ in the biceps at 5 months post surgery, stained with anti-NF200 to label regenerating axon (green), and anti-*α*-BT to label AchR clusters (red). In remodeled NMJs, thin axon terminals innervate some but not all fragmented AchR clusters (arrows in E, I). Neoformed NMJs are characterized by small AchR clusters innervated by thin axons that lack terminal arbors (asterisks in F, J). Quantification in K, 100 NMJs in each group, n = 4 mice; **: *P*<0.01, one-way ANOVA.

The formation of new NMJs is critical to functional recovery. We compared left (L, intact side) and right (R, surgery side) biceps brachii muscles 5 months after surgery. Muscle morphology did not display any evident anomaly in both genotypes (Fig. 11A). However, the R/L wet weight ratio was 97% in controls (n = 10) versus 80% in mutants (n = 10), a significant difference (Fig. 11B, *P*<0.01).We sampled 100 NMJs in 4 animals of each genotype and classified them as denervated, mature and immature (including remodeled and neoformed) [27], as illustrated in Fig. 11. All types of NMJs could be found in both groups (Fig. 11C–J). Compared to controls, the percentage of denervated NMJs was significantly higher (51% versus 14%), and that of mature and immature NMJs significantly lower in the mutant (24% versus 31%, 25% versus 55% respectively; Fig.11K; *P*<0.01).

**Figure 12:**
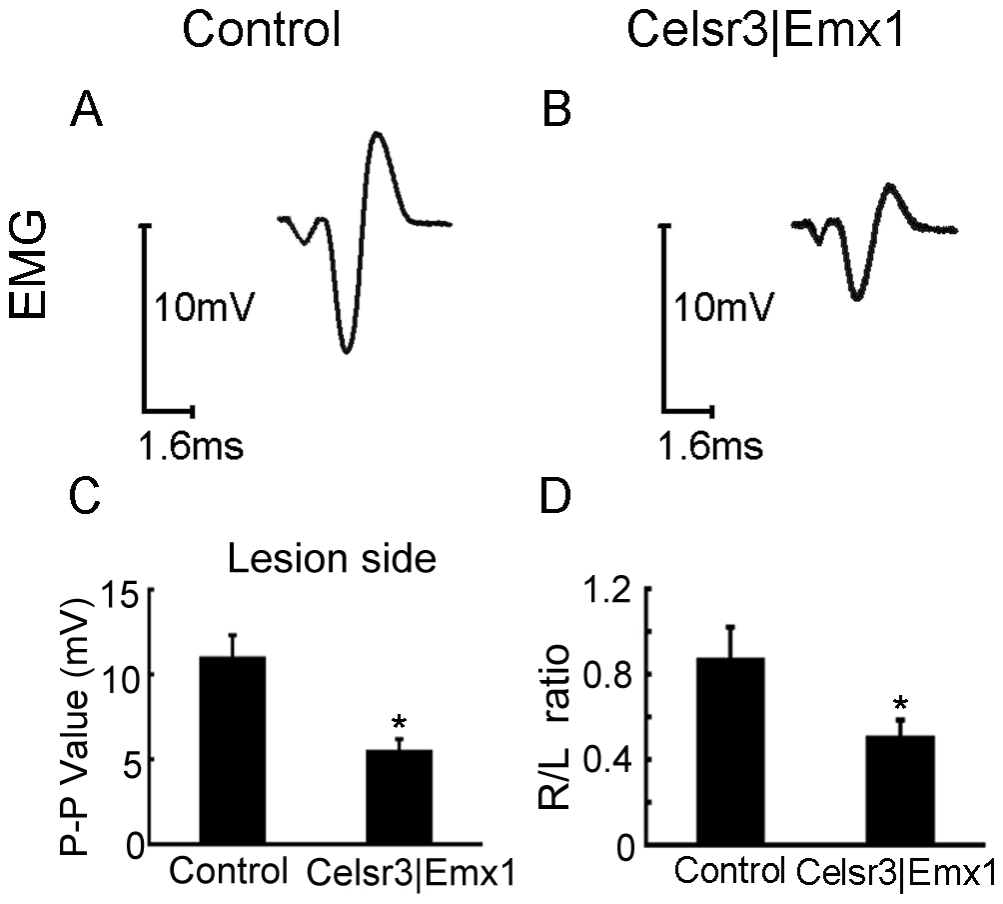
EMG amplitude in re-innervated muscles is smaller in *Celsr3|Emx1* than control mice. EMG was recorded in biceps 5 months post surgery, upon stimulation of the musculocutaneous nerve. On operated sides, the peak-to-peak amplitude is much reduced in the mutant (A, B, C). Simultaneously, EMG of biceps brachii was recorded on intact sides and the EMG peak ratio of the lesion to intact side (R/L) was calculated (D). The mean R/L ratio reaches about 88% in control mice versus 51% in mutant mice, showing a significant difference between two groups (D). *: *P*<0.05, *t*-test, n = 10 in each group.

To assess whether newly formed NMJs were functional, we recorded EMG of biceps brachii upon stimulation of musculocutaneous nerves. Stimuli–induced EMG in re-innervated muscles, particularly the peak-to-peak amplitude, was reduced in the mutant (Fig. 12A–C; *P*<0.05, n = 10 in each group). We also recorded EMG of biceps brachii on non operated sides and calculated the EMG peak ratio of the intact to lesion side (R/L), which was 88% in control versus 51% in mutant mice, a significant difference (Fig. 12C; *P*<0.05, n = 10 in each group).

## Discussion

In parallel to social and economic development, the number of SCI patients with brachial plexus injury (BPI) increases steadily, justifying the study of animal models [1], [34], [35]. The present work was aimed to assess the impact of absent corticospinal input on recovery from BPI, by using root avulsion and re-implantation in mice, and by comparing normal and mutant mice with congenital absence of CST. We show that the mutant mice with the absence of CST behave decreased anatomical and functional recovery of motor axons after root avulsion, but higher motoneuron survival.

Although motoneurons are located in the central nervous system, their axons have the potential to regenerate, presumably because they travel mostly in the periphery. Root avulsion with re-implantation has been used to test regeneration of motor axons, re-innervation of target muscles, and functional recovery, initially in cats [6], [36] and then in other animal models, as well as in patients [37], [38]. Root re-implantation provides a scaffold for regrowth of motoneuron axons, and fosters motoneuron survival [39], [40]. The biceps brachii is innervated mainly by C6 motoneurons. Avulsion of C5–C7 roots followed by C6 roots re-implantation therefore allows to focus on C6 motoneuron axon regeneration and biceps re-innervation [3], [30], [39], [41], [42].

Previous models to study the effects of cortical input on motoneuron function use cortical or CST lesions that disconnect a previously wired system in animals [17]–[19]. The genetic model used here is different in that the CST never develops in those animals, and that plasticity during development could palliate the absence of CST, therefore generating a non physiological situation. Plasticity during development is indeed supported by our evidence that the rubrospinal tract is increased in size in *Celsr3|Emx1* mice, whereas other descending tracts such as reticulo- or vestibulo spinal are unaffected (unpublished observations). Despite those intrinsic limitations, our genetic model has the advantage that no experimental lesion in cortex or CST is carried out, and therefore problems of interpretation related to the accuracy of surgical procedures, postsurgical inflammation and physiopathological and anatomical reactions to disconnections of other systems are eliminated.

Upon root avulsion and axotomy, motoneurons undergo a retrograde reaction and their phenotype switches from a transmitter to a regenerative state, which is reflected in gene expression profiles [43], [44]. For example, the expression of neurotrophic factor receptor genes changes [45], and this may explain why the local administration of neurotrophic factors fosters the regeneration of motor axons and functional rehabilitation [31], [46]–[48]. Changes in motoneuron morphology and gene expression after axotomy hamper quantification of motoneuron numbers using Nissl staining or immunohistochemistry. Inasmuch as such changes do not occur after removal of cortex or section of CST, they do not affect comparison of motoneuron numbers between control and *Celsr3|Emx1* mutant mice. But they clearly make it difficult to distinguish between decreased motoneuron numbers at 5 months (Fig. 4) and decreased marker expression, Nissl body concentration and/or shrinking of their soma. Since our observations concern comparison between mutants and controls, we believe that our main conclusion that mutant mice lacking CST have lower motoneuron axonal recovery yet higher survival following axotomy, are not or minimally affected by this issue.

When monitoring the dynamics of changes after root re-implantation, we found that a gradual recovery began at 3 weeks after surgery in control, indicating that plasticity changes in young adult mice proceed faster than in rats and cats [39], [42]. Root avulsion and re-implantation triggered motor axon regeneration, monitored by GAP-43 expression [49], and remyelination by Schwann cells, followed by MBP expression [50], as described in other settings. It also induced activation of microglia, which may contribute to neural repair by releasing neurotrophic factors and anti-inflammatory cytokines, while simultaneously damage cells after injury [51]. Those three events were generally more pronounced in control than in *Celsr3|Emx1* mutant animals. In addition, injury activates some genes expression at the early stage which may influence axonal regeneration. Higher increase of c-Jun in our observation in control mice probably contribute to better neural repair as seen in the morphology and function studies. Similarly, at five months post surgery, at the end of the plasticity period, behavioral tests as well as morphological analysis of peripheral nerve, of NMJ and EMG studies showed that recovery is more complete in control than in mutant mice. Somewhat paradoxically, the number of motoneurons that survived the procedure was higher in mutant than control animals. Neuronal survival is estimated by by the R/L ratio of spinal motoneuron number. In the mutant, there are less spinal motoneurons than in control mice, namely, the intact side (L) has less motoneurons in the mutant than in the control (Unpublished observation), and it is possible that remaining spinal motoneurons in the mutant are more resistant to injury. Higher expression of TrkB and BDNF and the lower microglial activation on injured spinal sides in the mutant than in the control may be contributing factors. In future, we plan to study this question further, particularly by comparing the expression of neurotrophic factors and their receptors at different time-points post surgery.

As mentioned in the Introduction, numerous studies proposed that corticospinal inputs regulate maturation and neural activities during the early development of the spinal cord, but not in adults, and cortical stimuli contribute to functional recovery after spinal cord injury. Although the mechanisms remain poorly defined and require further studies, our results provide evidence that corticospinal inputs probably influence spinal motoneuron axonal regeneration and functional recovery, and suggest that the manipulation of corticospinal transmission maybe a way to foster axonal repair after BPI.

## References

[1] Midha R (1997) Epidemiology of brachial plexus injuries in a multitrauma population. Neurosurgery 40: 1182–1188 discussion 1188–1189 9179891 10.1097/00006123-199706000-00014

[2] Koliatsos VE, Price WL, Pardo CA, Price DL (1994) Ventral root avulsion: an experimental model of death of adult motor neurons. J Comp Neurol 342: 35–44. 8207127 10.1002/cne.903420105

[3] Carlstedt T, Grane P, Hallin RG, Noren G (1995) Return of function after spinal cord implantation of avulsed spinal nerve roots. Lancet 346: 1323–1325. 7475770 10.1016/s0140-6736(95)92342-x

[4] Wu W, Han K, Li L, Schinco FP (1994) Implantation of PNS graft inhibits the induction of neuronal nitric oxide synthase and enhances the survival of spinal motoneurons following root avulsion. Exp Neurol 129: 335–339. 7525335 10.1006/exnr.1994.1176

[5] Carlstedt T, Anand P, Hallin R, Misra PV, Noren G, et al (2000) Spinal nerve root repair and reimplantation of avulsed ventral roots into the spinal cord after brachial plexus injury. J Neurosurg 93: 237–247. 10.3171/spi.2000.93.2.0237 11012054

[6] Midha R (2004) Nerve transfers for severe brachial plexus injuries: a review. Neurosurg Focus 16: E5. 10.3171/foc.2004.16.5.6 15174825

[7] Lemon RN (2008) Descending pathways in motor control. Annu Rev Neurosci 31: 195–218. 18558853 10.1146/annurev.neuro.31.060407.125547

[8] ten Donkelaar HJ, Lammens M, Wesseling P, Hori A, Keyser A, et al (2004) Development and malformations of the human pyramidal tract. J Neurol 251: 1429–1442. 15645341 10.1007/s00415-004-0653-3

[9] Terashima T (1995) Anatomy, development and lesion-induced plasticity of rodent corticospinal tract. Neurosci Res 22: 139–161. 7566696 10.1016/0168-0102(95)00895-9

[10] Joosten EA, Schuitman RL, Vermelis ME, Dederen PJ (1992) Postnatal development of the ipsilateral corticospinal component in rat spinal cord: a light and electron microscopic anterograde HRP study. J Comp Neurol 326: 133–146. 1479066 10.1002/cne.903260112

[11] Oudega M, Varon S, Hagg T (1994) Distribution of corticospinal motor neurons in the postnatal rat: quantitative evidence for massive collateral elimination and modest cell death. J Comp Neurol 347: 115–126. 7798376 10.1002/cne.903470109

[12] Eyre JA (2007) Corticospinal tract development and its plasticity after perinatal injury. Neurosci Biobehav Rev 31: 1136–1149. 18053875 10.1016/j.neubiorev.2007.05.011

[13] Dekkers J, Greensmith L, Navarrete R (2002) Changes in the expression of parvalbumin immunoreactivity in the lumbar spinal cord of the rat following neonatal nerve injury. Dev Neurosci 24: 283–293. 12457066 10.1159/000066742

[14] Gibson CL, Clowry GJ (1999) Retraction of muscle afferents from the rat ventral horn during development. Neuroreport 10: 231–235. 10203314 10.1097/00001756-199902050-00006

[15] Kudo N, Yamada T (1987) Morphological and physiological studies of development of the monosynaptic reflex pathway in the rat lumbar spinal cord. J Physiol 389: 441–459. 2824763 10.1113/jphysiol.1987.sp016665 PMC1192089

[16] Chakrabarty S, Shulman B, Martin JH (2009) Activity-dependent codevelopment of the corticospinal system and target interneurons in the cervical spinal cord. J Neurosci 29: 8816–8827. 19587289 10.1523/JNEUROSCI.0735-09.2009 PMC3849701

[17] Clowry GJ, Fallah Z, Arnott G (1997) Developmental expression of parvalbumin by rat lower cervical spinal cord neurones and the effect of early lesions to the motor cortex. Brain Res Dev Brain Res 102: 197–208. 9352102 10.1016/s0165-3806(97)00098-9

[18] Clowry GJ, Davies BM, Upile NS, Gibson CL, Bradley PM (2004) Spinal cord plasticity in response to unilateral inhibition of the rat motor cortex during development: changes to gene expression, muscle afferents and the ipsilateral corticospinal projection. Eur J Neurosci 20: 2555–2566. 15548199 10.1111/j.1460-9568.2004.03713.x

[19] Gibson CL, Arnott GA, Clowry GJ (2000) Plasticity in the rat spinal cord seen in response to lesions to the motor cortex during development but not to lesions in maturity. Exp Neurol 166: 422–434. 11085907 10.1006/exnr.2000.7511

[20] Han Q, Feng J, Qu Y, Ding Y, Wang M, et al (2013) Spinal cord maturation and locomotion in mice with an isolated cortex. Neuroscience 253: 235–244. 24012835 10.1016/j.neuroscience.2013.08.057

[21] Brus-Ramer M, Carmel JB, Chakrabarty S, Martin JH (2007) Electrical stimulation of spared corticospinal axons augments connections with ipsilateral spinal motor circuits after injury. J Neurosci 27: 13793–13801. 18077691 10.1523/JNEUROSCI.3489-07.2007 PMC6673617

[22] Carmel JB, Berrol LJ, Brus-Ramer M, Martin JH (2010) Chronic electrical stimulation of the intact corticospinal system after unilateral injury restores skilled locomotor control and promotes spinal axon outgrowth. J Neurosci 30: 10918–10926. 20702720 10.1523/JNEUROSCI.1435-10.2010 PMC2929360

[23] Carmel JB, Kimura H, Berrol LJ, Martin JH (2013) Motor cortex electrical stimulation promotes axon outgrowth to brain stem and spinal targets that control the forelimb impaired by unilateral corticospinal injury. Eur J Neurosci 37: 1090–1102. 23360401 10.1111/ejn.12119 PMC3618589

[24] Bunday KL, Perez MA (2012) Motor recovery after spinal cord injury enhanced by strengthening corticospinal synaptic transmission. Curr Biol 22: 2355–2361. 23200989 10.1016/j.cub.2012.10.046 PMC3742448

[25] Zhou L, Bar I, Achouri Y, Campbell K, De Backer O, et al (2008) Early forebrain wiring: genetic dissection using conditional Celsr3 mutant mice. Science 320: 946–949. 18487195 10.1126/science.1155244 PMC2746700

[26] Bertelli JA, Mira JC (1993) Behavioral evaluating methods in the objective clinical assessment of motor function after experimental brachial plexus reconstruction in the rat. J Neurosci Methods 46: 203–208. 8483313 10.1016/0165-0270(93)90068-3

[27] Huze C, Bauche S, Richard P, Chevessier F, Goillot E, et al (2009) Identification of an agrin mutation that causes congenital myasthenia and affects synapse function. Am J Hum Genet 85: 155–167. 19631309 10.1016/j.ajhg.2009.06.015 PMC2725239

[28] Chomiak T, Hu B (2009) What is the optimal value of the g-ratio for myelinated fibers in the rat CNS? A theoretical approach. PLoS One 4: e7754. 19915661 10.1371/journal.pone.0007754 PMC2771903

[29] Li L, Oppenheim RW, Lei M, Houenou LJ (1994) Neurotrophic agents prevent motoneuron death following sciatic nerve section in the neonatal mouse. J Neurobiol 25: 759–766. 8089654 10.1002/neu.480250702

[30] Gu HY, Chai H, Zhang JY, Yao ZB, Zhou LH, et al (2005) Survival, regeneration and functional recovery of motoneurons after delayed reimplantation of avulsed spinal root in adult rat. Exp Neurol 192: 89–99. 15698622 10.1016/j.expneurol.2004.10.019

[31] Chu TH, Wu W (2009) Neurotrophic factor treatment after spinal root avulsion injury. Cent Nerv Syst Agents Med Chem 9: 40–55. 20021337 10.2174/187152409787601914

[32] Raivich G, Bohatschek M, Da Costa C, Iwata O, Galiano M, et al (2004) The AP-1 transcription factor c-Jun is required for efficient axonal regeneration. Neuron 43: 57–67. 15233917 10.1016/j.neuron.2004.06.005

[33] Wu W, Li Y, Schinco FP (1994) Expression of c-jun and neuronal nitric oxide synthase in rat spinal motoneurons following axonal injury. Neurosci Lett 179: 157–161. 7531312 10.1016/0304-3940(94)90958-x

[34] Faglioni W Jr, Siqueira MG, Martins RS, Heise CO, Foroni L (2014) The epidemiology of adult traumatic brachial plexus lesions in a large metropolis. Acta Neurochir (Wien) 156(5): 1025–1028. 24318512 10.1007/s00701-013-1948-x

[35] Dorsi MJ, Hsu W, Belzberg AJ (2010) Epidemiology of brachial plexus injury in the pediatric multitrauma population in the United States. J Neurosurg Pediatr 5: 573–577. 20515329 10.3171/2010.3.PEDS09538

[36] Cullheim S, Carlstedt T, Linda H, Risling M, Ulfhake B (1989) Motoneurons reinnervate skeletal muscle after ventral root implantation into the spinal cord of the cat. Neuroscience 29: 725–733. 2739906 10.1016/0306-4522(89)90144-9

[37] Carlstedt T (2009) Nerve root replantation. Neurosurg Clin N Am 20: 39–50, vi. 10.1016/j.nec.2008.07.020 19064178

[38] Fournier HD, Mercier P, Menei P (2005) Repair of avulsed ventral nerve roots by direct ventral intraspinal implantation after brachial plexus injury. Hand Clin 21: 109–118. 15668071 10.1016/j.hcl.2004.09.001

[39] Gu HY, Chai H, Zhang JY, Yao ZB, Zhou LH, et al (2004) Survival, regeneration and functional recovery of motoneurons in adult rats by reimplantation of ventral root following spinal root avulsion. Eur J Neurosci 19: 2123–2131. 15090039 10.1111/j.0953-816X.2004.03295.x

[40] Hoang TX, Havton LA (2006) A single re-implanted ventral root exerts neurotropic effects over multiple spinal cord segments in the adult rat. Exp Brain Res 169: 208–217. 16273401 10.1007/s00221-005-0137-4

[41] Chai H, Wu W, So KF, Yip HK (2000) Survival and regeneration of motoneurons in adult rats by reimplantation of ventral root following spinal root avulsion. Neuroreport 11: 1249–1252. 10817601 10.1097/00001756-200004270-00021

[42] Hoffmann CF, Marani E, van Dijk JG, vd Kamp W, Thomeer RT (1996) Reinnervation of avulsed and reimplanted ventral rootlets in the cervical spinal cord of the cat. J Neurosurg 84: 234–243. 8592226 10.3171/jns.1996.84.2.0234

[43] Navarro X, Vivo M, Valero-Cabre A (2007) Neural plasticity after peripheral nerve injury and regeneration. Prog Neurobiol 82: 163–201. 17643733 10.1016/j.pneurobio.2007.06.005

[44] Risling M, Ochsman T, Carlstedt T, Linda H, Plantman S, et al (2011) On acute gene expression changes after ventral root replantation. Front Neurol 1: 159. 21228913 10.3389/fneur.2010.00159 PMC3018771

[45] Hammarberg H, Piehl F, Risling M, Cullheim S (2000) Differential regulation of trophic factor receptor mRNAs in spinal motoneurons after sciatic nerve transection and ventral root avulsion in the rat. J Comp Neurol 426: 587–601. 11027401 10.1002/1096-9861(20001030)426:4<587::aid-cne7>3.0.co;2-r

[46] Novikov L, Novikova L, Kellerth JO (1997) Brain-derived neurotrophic factor promotes axonal regeneration and long-term survival of adult rat spinal motoneurons in vivo. Neuroscience 79: 765–774. 9219940 10.1016/s0306-4522(96)00665-3

[47] Blits B, Carlstedt TP, Ruitenberg MJ, de Winter F, Hermens WT, et al (2004) Rescue and sprouting of motoneurons following ventral root avulsion and reimplantation combined with intraspinal adeno-associated viral vector-mediated expression of glial cell line-derived neurotrophic factor or brain-derived neurotrophic factor. Exp Neurol 189: 303–316. 15380481 10.1016/j.expneurol.2004.05.014

[48] Zhou LH, Wu W (2006) Survival of injured spinal motoneurons in adult rat upon treatment with glial cell line-derived neurotrophic factor at 2 weeks but not at 4 weeks after root avulsion. J Neurotrauma 23: 920–927. 16774476 10.1089/neu.2006.23.920

[49] Harding DI, Greensmith L, Mason M, Anderson PN, Vrbova G (1999) Overexpression of GAP-43 induces prolonged sprouting and causes death of adult motoneurons. Eur J Neurosci 11: 2237–2242. 10383612 10.1046/j.1460-9568.1999.00640.x

[50] Forghani R, Garofalo L, Foran DR, Farhadi HF, Lepage P, et al (2001) A distal upstream enhancer from the myelin basic protein gene regulates expression in myelin-forming schwann cells. J Neurosci 21: 3780–3787. 11356866 10.1523/JNEUROSCI.21-11-03780.2001 PMC6762685

[51] Gomes-Leal W (2012) Microglial physiopathology: how to explain the dual role of microglia after acute neural disorders? Brain Behav 2: 345–356. 22741103 10.1002/brb3.51 PMC3381634

